# A multicenter phase II study of S-1 for gemcitabine-refractory biliary tract cancer

**DOI:** 10.1007/s00280-013-2106-0

**Published:** 2013-03-24

**Authors:** Eiichiro Suzuki, Masafumi Ikeda, Takuji Okusaka, Shoji Nakamori, Shinichi Ohkawa, Tatsuya Nagakawa, Narikazu Boku, Hiroaki Yanagimoto, Tosiya Sato, Junji Furuse

**Affiliations:** 1 Department of Internal Medicine, Medical Oncology, Kyorin University School of Medicine, 6-20-2, Shinkawa, Mitaka, Tokyo, 181-8611 Japan; 2 Division of Hepatobiliary and Pancreatic Medical Oncology, National Cancer Center Hospital East, Kashiwa, Japan; 3 Division of Hepatobiliary and Pancreatic Oncology, National Cancer Center Hospital, Tokyo, Japan; 4 Department of Surgery, National Hospital Organization Osaka National Hospital, Osaka, Japan; 5 Division of Hepatobiliary and Pancreatic Medical Oncology, Kanagawa Cancer Center, Yokohama, Japan; 6 Department of Gastroenterology, Sapporo Kosei General Hospital, Sapporo, Japan; 7 Division of Gastrointestinal Oncology, Shizuoka Cancer Center, Shizuoka, Japan; 8 Department of Surgery, Kansai Medical University, Hirakata, Japan; 9 Department of Biostatistics, Kyoto University School of Public Health, Kyoto, Japan

**Keywords:** Biliary tract cancer, S-1, Gemcitabine, Refractory

## Abstract

**Purpose:**

Gemcitabine (GEM)-based chemotherapy has been used worldwide as the first-line treatment for advanced biliary tract cancer (BTC). However, no standard regimens have been established yet for patients with GEM-refractory BTC. A previous phase II trial of S-1 as a first-line treatment in patients with advanced BTC revealed promising activity of this drug. The present study was conducted to evaluate the efficacy and safety of S-1 in patients with GEM-refractory BTC.

**Methods:**

The subjects were patients with pathologically proven BTC who had shown disease progression while receiving GEM-based chemotherapy. Each treatment cycle consisted of administration of S-1 orally at the dose of 40 mg/m^2^ twice daily for 28 days, followed by a rest period of 14 days. The primary endpoint of this study was objective response, and the secondary endpoints were the toxicity, progression-free survival (PFS), and overall survival (OS).

**Results:**

Forty patients were assessed for efficacy and safety from 8 hospitals in Japan between June 2007 and September 2008. There were 3 cases of confirmed partial response (7.5 %) and 22 patients (55 %) of stable disease. The median PFS and OS were 2.5 and 6.8 months, respectively. Toxicity was generally mild, and the most common grade 3 or 4 toxicities were anorexia (10.0 %), anemia (7.5 %), mucositis (7.5 %), hypoalbuminemia (5.0 %), and pneumonia (5.0 %). There were no treatment-related deaths.

**Conclusions:**

Monotherapy with S-1 was well tolerated, but showed modest efficacy in patients with GEM-refractory BTC.

## Introduction

Biliary tract cancer (BTC), while being relatively uncommon in Western countries, is a common cause of death in Japan, Korea, and Chile [1, 2]. Resection offers the only chance for cure of the disease. However, the resectability rate is generally low because the disease is generally diagnosed at advanced stage. Moreover, the majority of patients with resected BTC eventually develop recurrence(s) [3]. Therefore, systemic chemotherapy has been the mainstay of the treatment for most patients with BTC.

To date, various drugs have been investigated for the treatment of BTC. Among them, gemcitabine (GEM)-based regimens have exhibited moderate activity against BTC [4]. Recently, in a randomized phase III study comparing combination chemotherapy of GEM and cisplatin with GEM monotherapy (UK ABC-02 study), combination chemotherapy yielded survival benefit over GEM monotherapy, with median survival times of 11.7 months in the former arm versus 8.3 months in the latter arm (*P* = 0.002) [5]. This study was the first large-scale randomized trial conducted in patients with BTC, and the combination chemotherapy of GEM and cisplatin has been established as standard chemotherapy for patients with advanced BTC. A randomized phase II study conducted in Japan also showed similar results [6]. Despite these progresses in chemotherapy, however, the survival is still not satisfactory. In many other cancers, the second-line chemotherapy contributes to prolongation of survival. Thus, there is an urgent need to develop effective second-line chemotherapies for patients with BTC. To date, however, second-line chemotherapy for patients with BTC refractory to treatment with GEM-based regimens has not been fully examined.

S-1 (Taiho Pharmaceutical Co., Ltd., Tokyo, Japan) is a novel orally administered anticancer drug consisting of a combination of tegafur (FT), 5-chloro-2,4-dihydroxypyridine (CDHP), and oteracil potassium (Oxo) in a molar concentration ratio of 1:0.4:1 [7]. CDHP is a competitive inhibitor of dihydropyrimidine dehydrogenase, which is involved in the degradation of 5-FU, and acts to maintain efficacious concentrations of 5-FU in the plasma and tumor tissues [8]. Oxo, a competitive inhibitor of orotate phosphoribosyl transferase, inhibits the phosphorylation of 5-FU in the gastrointestinal tract, thereby serving to reduce the serious gastrointestinal toxicity associated with 5-FU treatment [9]. The antitumor effect of S-1 has already been demonstrated in a variety of solid tumors [10]. A recent late phase II study conducted to evaluate the efficacy of S-1 in chemo-naive advanced BTC patients demonstrated promising results, with a response rate of 35.0 % and a favorable toxicity profile [11]. Therefore, we conducted a phase II study to investigate the efficacy and safety of S-1 in patients with GEM-refractory BTC.

## Patients and methods

### Patients

The inclusion criteria for this study were as follows: (1) histologically proven BTC, (2) progressive disease (PD) during the GEM-based first-line chemotherapy, (3) 20–79 years of age, (4) Eastern Cooperative Oncology Group performance status (PS) 0–2, 5) more than 3 weeks from the last administration of the previous chemotherapy, 6) adequate bone marrow functions (white blood cell count ≥3,000/mm^3^, neutrophil count ≥1,500/mm^3^, platelet count ≥100,000/mm^3^, and hemoglobin ≥9.0 g/dL), (7) adequate renal function (serum creatinine ≤1.5 mg/dL), and (8) adequate liver function (serum total bilirubin ≤2.0 mg/dL, serum transaminases ≤2.5 times the upper limit of the respective normal ranges). Patients who had obstructive jaundice or liver metastasis were considered to be eligible if their serum transaminase levels could be reduced to within 5 times the upper limit of normal by biliary drainage. The exclusion criteria were as follows: (1) under regular treatment with phenytoin, warfarin, or flucytosine (2) history of chemotherapy with fluorinated pyrimidine, (3) severe mental disorder, active infection, ileus, interstitial pneumonia or pulmonary fibrosis, uncontrolled diabetes mellitus, heart failure, renal failure, active gastric or duodenal ulcer, massive pleural or abdominal effusion, and brain metastasis, (4) active concomitant malignancy, and (5) pregnant/lactating women. Written informed consent was obtained from all of the patients. This study was conducted with the approval of the institutional review board at all the participating hospitals. The study is registered with the UMIN Clinical Trials Registry as UMIN000000919.

### Treatment

S-1 was administered orally at the dose of 40 mg/m^2^ twice daily, after meals. Three initial doses were set according to the body surface area (BSA), as follows: BSA < 1.25 m^2^, 80 mg/day; 1.25 m^2^ ≤ BSA < 1.50 m^2^, 100 mg/day; 1.50 m^2^ ≤ BSA, 120 mg/day. S-1 was administered for 28 days, followed by a 14-day rest period. The treatment cycle was repeated until the detection of disease progression, appearance of unacceptable toxicities, or patient’s refusal.

If any grade 3 or more severe hematologic, or grade 2 or more severe non-hematologic toxicity occurred, administration of S-1 was either temporarily discontinued until the toxicity recovered to grade 1 or less, and the dose of S-1 was reduced by 20 mg/day in the next treatment cycle. If no toxicity occurred, the scheduled rest period was shortened to 7 days (4-week cycle), or the dose was gradually escalated in the next course (maximum dose, 150 mg/day), or both were permitted according to the judgment of the individual physicians. In a case of the course delay more than 28 days due to toxicity, the protocol treatment was discontinued. Patients were not allowed to receive concomitant radiation therapy, chemotherapy, or hormonal therapy during the study.

### Response and toxicity evaluation

The response after each course was evaluated according to the Response Evaluation Criteria in Solid Tumors. Physical examinations, complete blood cell counts, biochemistry tests, and urinalyses were assessed at least once every 2 weeks. Adverse events were evaluated according to the National Cancer Institute Common Toxicity Criteria, version 3.0.

### Statistical analysis

The primary endpoint of this study was objective response rate. The secondary endpoints were toxicity, progression-free survival (PFS), and overall survival (OS). The target number of patients in this study was determined using a Southwest Oncology Group’s standard [12, 13]. The null hypothesis was that the overall response rate would be ≤5 %, and the alternative hypothesis was that the overall response rate would be ≥15 %, the α level was 5 % (one tailed), and the power was 10 % (one tailed). The alternative hypothesis was established based on the data from our previous studies of first-line treatment [14, 15]. Interim analysis was planned when 20 patients were enrolled. If none of the first 20 patients showed a partial or complete response (CR), the study itself was to be discontinued. If a response was detected in the first 20 patients, 20 patients were added in the second stage if the lower limit of the 90 % confidence interval (CI) exceeded the 5 % threshold (objective response in ≥7 of the 40 patients), S-1 would be judged to be effective, and we would proceed to the next large-scale study. The PFS was calculated from the date of study entry to the date of documented disease progression or death due to any cause. The OS was calculated from the date of study entry to the date of death or the date of last follow-up. The median probability of the survival period and PFS were estimated using the Kaplan–Meier method. The relative dose intensity of S-1 was calculated according to the Hryniuk method [16].

## Results

A total of 41 patients were enrolled in this study. Of these 41 patients, one patient was excluded on account of the rapid clinical deterioration before the first administration of S-1, and the remaining 40 patients were assessed. The patient characteristics are shown in Table 1. Of the 40 patients, 35 received GEM monotherapy and the remaining 5 received combined therapy with GEM plus cisplatin as the first-line chemotherapy. As the best response to the first-line chemotherapy, one patient showed CR, two patients showed partial response (PR), 19 patients showed stable disease (SD), and the remaining 14 patients showed PD. Progress disease was observed in all patients during the first-line chemotherapy. The median time to progression during this first-line chemotherapy was 4.3 months (range 0.9–17.8).

**Table 1:** Patient characteristics

Characteristics	Number of patients (%)
Age (years) [median (range)]	67 (35–78)
Sex	
Male	26 (65)
Female	14 (35)
Performance status	
0	18 (45)
1	20 (50)
2	2 (5)
Primary tumor site	
Intrahepatic bile duct	10 (25)
Extrahepatic bile duct	15 (38)
Gall bladder	14 (35)
Ampulla of Vater	1 (3)
Extent of disease	
Locally advanced	3 (8)
Metastatic	37 (92)
Cancer involvement	
Liver	25 (63)
Lymph node	18 (45)
Peritoneal dissemination	4 (2)
Lung	8 (20)
Biliary drainage (+)	21 (53)
Prior surgical resection (+)	20 (50)
Prior chemotherapy	
Gemcitabine	35 (88)
Gemcitabine + cisplatin	5 (13)
Carcinoembryonic antigen (ng/mL) (median [range])	5 (1–1,837)
Carbohydrate antigen 19–9 (U/mL) (median [range])	751 (3–71,900)

### Treatment

A total of 92 courses were administered to the 40 patients, with a median of two courses per patient (range 1–12). The relative dose intensity of S-1 was 97.3 %. The reasons for discontinuation of treatment were radiologically confirmed PD (31 patients), clinically confirmed PD without radiological confirmation (5 patients), unacceptable toxicities (two patients; one patient the course delay more than 28 days due to continuing grade 2 nausea, and the other patient grade 4 leukoencephalopathy), patient’s request to withdraw from the study (one patient), or surgical resection because of PR (one patient).

### Toxicity

Forty patients were assessable for adverse events. The treatment-related adverse events are shown in Table 2. Toxicity was generally mild, and the major grade 3 or 4 toxicities were anorexia (10.0 %), anemia (7.5 %), mucositis (7.5 %), hypoalbuminemia (5.0 %), and pneumonia (5.0 %). One patient developed grade 4 leukoencephalopathy, but recovered with just observation. Although two patients died due to rapid disease progression within 4 weeks of discontinuation of the treatment, no treatment-related deaths were observed.

**Table 2:** Treatment-related adverse events (*n* = 40): worst grade reported during the treatment period

Toxicity grade	1	2	3	4	1–4 (%)	3/4 (%)
Hematological toxicity						
Leukopenia	7	0	0	1	8 (20)	1 (3)
Neutropenia	2	1	0	1	4 (10)	1 (3)
Anemia	4	8	3	0	15 (38)	3 (8)
Thrombocytopenia	9	2	1	0	12 (30)	1 (3)
Non-hematological toxicity						
Nausea	6	4	0	0	10 (25)	0 (0)
Vomiting	3	1	0	0	4 (10)	0 (0)
Anorexia	10	5	5	0	20 (50)	5 (13)
Fatigue	9	6	1	0	16 (40)	1 (3)
Diarrhea	2	3	2	0	7 (18)	2 (5)
Rash	2	1	0	0	3 (8)	0 (0)
Decreased serum albumin level	6	2	2	0	10 (8)	2 (5)
Elevated serum AST	5	1	0	0	6 (15)	0 (0)
Elevated serum ALT	2	0	0	0	2 (5)	0 (0)
Elevated serum total bilirubin	3	1	1	0	5 (13)	1 (3)
Elevated serum creatinine	1	0	0	0	1 (3)	0 (0)
Encephalopathy	0	0	0	1	1 (3)	1 (3)
Mucositis	6	0	3	0	9 (23)	3 (8)
Biliary tract infection	0	1	1	0	2 (5)	1 (3)
Colitis	0	1	1	0	2 (5)	1 (3)
Taste disturbance	1	1	0	0	2 (5)	0 (0)
Pigmentation	4	1	0	0	5 (13)	0 (0)
Abdominal pain	6	2	0	0	8 (20)	0 (0)

*AST* aspartate aminotransferase, *ALT* alanine aminotransferase

### Efficacy

Forty patients were assessed for response. The responses are shown in Table 3. There was no case of CR; however, 3 patients [2 patients with intrahepatic cholangiocarcinoma (IHC) and one patient with gall bladder carcinoma (GBC)] showed PR. Twenty-two patients showed SD, and 15 patients showed PD. The overall response rate was 7.5 % (95 % CI 1.6–20.4 %; 90 % CI 2.1–18.3 %), and the disease control rate was 62.5 % (95 % CI 45.8–77.2 %).

**Table 3:** Response rate and tumor control rate in patients with gall bladder carcinoma, intrahepatic and extrahepatic cholangiocarcinoma, and ampulla of Vater cancer

Outcome	Total (*n* = 40)	GBC (*n* = 14)	IHC (*n* = 10)	EHC (*n* = 15)	AVC (*n* = 1)
CR	0	0	0	0	0
PR	3	1	2	0	0
SD	22	5	7	9	1
PD	15	8	1	6	0
Response rate (%)	7.5	7.1		7.7	
Disease control rate (%) (CR + PR +SD)	57.5	42.9		70.8	

*GBC* gall bladder carcinoma, *EHC* extrahepatic cholangiocarcinoma, *IHC* intrahepatic cholangiocarcinoma, *AVC* ampulla of Vater cancer

**Fig. 1:**
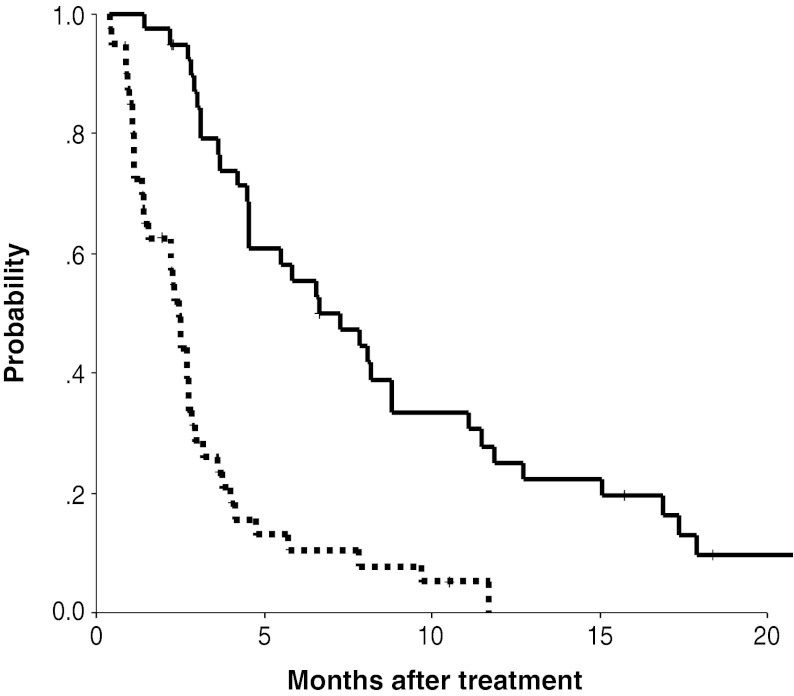
Progression-free survival (*dash line*) and overall survival (*solid line*) *curves* of patients with gemcitabine-refractory biliary tract cancer receiving systemic chemotherapy with S-1 (*n* = 40). The median progression-free survival and overall survival were 2.5 and 6.8 months, respectively. *Tick marks* indicate censored cases

Table 3 also shows the tumor responses according to the primary tumor site. The overall response rate and disease control rate in the GBC group (*n* = 14) were 7.1 and 42.9 %, respectively. Those with the primary tumors at other sites (IHC *n* = 10, extrahepatic cholangiocarcinoma (EHC) *n* = 15, and ampulla of Vater cancer (AVC) *n* = 1) were 7.7 and 70.8 %, respectively. The median PFS and OS of the 40 patients were 2.5 and 6.8 months, respectively (Fig. 1). The median PFS and OS of the patients with GBC were 1.4 and 4.7 months, respectively, and those of the patients with the primary tumor at other sites (IHC, EHC, and AVC) were 2.5 and 7.5 months, respectively.

## Discussion

The primary endpoint of this study was response rate. S-1 yielded a response rate of 7.5 % in the patients with GEM-refractory BTC. The lower 90 % confidence limit of the response rate, 2.1 %, was not above the null hypothesis (5 %), and hence, we did not consider that S-1 was effective.

However, since the disease control rate was 65.2 %, we concluded that the treatment showed modest efficacy. At present, several reports of clinical trials of second-line treatment are available (Table 4) [17–23]. The current study results were comparable to those of previous studies, except for another phase II trial of S-1 conducted on a small number of patients [21], in which the response rate ranged from 0 to 12.9 %.

**Table 4:** Clinical trials of second-line treatments for patients with advanced biliary tract cancer

Reference	Regimen	Number of patients	Response rate (%)	Median PFS/TTP (months)	Median OS (months)
Lee et al. [17]	5FU + ADR + MMC	31 (16)^a^	12.9	2.3	6.7
Oh et al. [18]	Gemcitabine	32	6.9	1.6	4.1
Pino et al. [19]	Capecitabine + celecoxib	35 (5)^a^	9	4.2	4.8
Sasaki et al. [20]	Gemcitabine + cisplatin	20	0	3.6	5.9
Sasaki et al. [21]	S-1	22	22.7	5.5	8.0
Yi et al. [22]	Sunitinib	56	8.9	1.7	12.9
Chiorean et al. [23]	Erlotinib + ADR	11	0	4.7	5.7
Current study	S-1	40	7.5	2.5	6.8

*PFS* progression-free survival, *TTP* time to progression, *OS* overall survival, *ADR* adriamycin, *MMC* mitomycin

^a^The number of patients includes both patients with pancreatic cancer and biliary tract cancer. The number in parentheses indicates the number of biliary tract cancer patients

In the current study, the median PFS and OS were 2.3 and 6.8 months, respectively. As indicated by several previous reports, BTC is a heterogeneous group, and the prognosis of cholangiocarcinoma and AVC is generally better than that of GBC [3, 24]. In this study, the disease control rate (PR + SD), PFS, and OS in the GBC group were worse than those with other primary sites.

With regard to toxicity, the results were similar to those observed during the previous first-line treatment with S-1 in chemo-naive patients with BTC [11, 25]. In addition, comparing this study with other clinical trials [17–23], we conclude second-line treatment with S-1 was well tolerated. Considering its safety and convenience, the drug can be used for treatment in the outpatient setting.

Based on the results of a randomized phase III trial of GEM + cisplatin versus GEM, the GEM + cisplatin regimen came to be recognized as standard first-line therapy for BTC. In regard to the second-line treatment, discrepant results were obtained between the randomized trials performed in the UK and those performed in Japan [26]. In the UK, the treatment for the majority of cases after disease progression in the first line was best supportive care, with only 17 % of the patients receiving further chemotherapy, mostly 5-FU-based chemotherapy. On the other hand, a much higher proportion of Japanese patients received second-line treatment, mainly with S-1 (75 % of patients). Despite this difference in the proportion of patients receiving second-line treatment, which might be expected to improve the survival after failure the first-line chemotherapy in Japanese trial patients as compared with that in the UK trial patients, the OS appeared to be very similar between the two trials. Thus, survival benefit of the second-line chemotherapy has not been confirmed. There is an urgent need to establish an effective second-line treatment(s) to improve the survival. The results of this study can serve as a reliable database for further studies on second-line treatment for BTC. The efficacy of second-line treatment should be assessed prospectively within randomized controlled trials.

In conclusion, S-1, administered as single-agent chemotherapy, was well tolerated, but showed modest efficacy in patients with GEM-refractory BTC.
